# Transcriptome Analysis Unravels CD4^+^ T-Cell and Treg-Cell Differentiation in Ovarian Cancer

**DOI:** 10.3390/biom15091241

**Published:** 2025-08-27

**Authors:** Baoyi Shao, Bo Sun, Zhongdang Xiao

**Affiliations:** State Key Laboratory of Bioelectronics, School of Biological Science and Medical Engineering, Southeast University, Nanjing 210096, China

**Keywords:** CD4^+^ T cell, Treg cell, ovarian cancer, tumor immune microenvironment, transcriptome

## Abstract

Background: Ovarian cancer ranks as the fifth leading cause of cancer-related mortality among women worldwide. Owing to its insidious onset and lack of early symptoms, over 70% of patients are diagnosed at advanced stages. Methods: This study provides a comprehensive transcriptomic analysis of tumor-infiltrating CD4^+^ T cells in ovarian cancer, highlighting regulatory T cells (Tregs) as the dominant subset. By integrating seven multicenter ovarian cancer single-cell RNA-seq datasets, a robust metadata resource was created for detailed Treg investigation. Using the BayesPrism algorithm, Treg scores from TCGA bulk RNA-seq data enabled patient stratification into high and low Treg groups. These findings were further validated through survival analyses across five independent bulk RNA-seq cohorts. We experimentally validated the inhibitory role of Tregs in modulating CD8^+^ T-cell activity in ovarian cancer. Results: We conducted an in-depth investigation into the clustering patterns, differentiation trajectories, intercellular interactions, and enrichment profiles of tumor-infiltrating T cells in ovarian cancer. Among the seven functionally defined subclusters (C1–C7), we delineated two distinct “terminal states” of CD4^+^ T-cell differentiation: FOXP3^+^ regulatory T cells and STMN1^+^ proliferative T cells. The OCSCDs dataset comprises seven datasets totaling 137,648 single cells. Using the TCGA dataset, we quantified the proportion of tumor-infiltrating regulatory T cells (Tregs) in OCSCDs through the BayesPrism algorithm and performed survival analyses across five independent bulk RNA-seq datasets from different platforms. Conclusions: Our results establish a framework for studying Treg biology in ovarian cancer and these cells may be become an important point in the field of immunotherapy.

## 1. Introduction

Ovarian cancer (OC), ranking as the third most prevalent gynecologic malignancy, accounts for approximately 314,000 new cases and 207,000 deaths annually worldwide, representing the highest fatality rate among gynecological cancers [1,2]. Approximately 70% of women are diagnosed with ovarian cancer at an advanced stage (stage III or IV), often accompanied by tumor metastasis to the peritoneal cavity [3]. The standard treatments for OC primarily consists of debulking surgery followed by combination platinum-based chemotherapy [4]. Despite of these standard-of-care therapies, the 5-year overall survival rate for patients diagnosed with advanced disease is still below 25% [5]. A significant number of patients develop recurrence after an initial response to conventional treatment, and resistance to chemotherapy was developed in the large proportion of them, which leads to death. Therefore, finding out new targeted therapies is the key point to improve the survival rate of OC patients.

An increasing number of studies have demonstrated that OC is immunogenic cancer [6]. Our understanding of the molecular and genetic alterations within the ovarian cancer tumor microenvironment (TME) has increased significantly. Accordingly, many immunotherapies target TME, aiming to overcome challenges posed by its strong immunosuppression [7]. Currently, immunotherapeutic approaches for OC primarily include cancer vaccines, adoptive cell therapy (ACT), immune checkpoint blockade (ICI), and oncolytic virus [8]. Tumors responsive to ICIs—termed “hot tumors”—are characterized by the degree of T-cell infiltration for this responsiveness. In contrast, “cold tumors” generally do not respond to ICIs, as they exhibit low T-cell infiltration [9,10]. One of the major challenges in immunotherapy is the generation and maintenance of functional, tumor-specific T cells. Although certain immunotherapies for OC, such as Poly (ADP-ribose) polymerase inhibitors (PARP inhibitors), have shown promising results in clinical trials [11], only a small proportion of OC patients are helped by them, and these patients eventually experience disease recurrence or metastasis. Hence, one can see that the research of tumor infiltrating T cells is particularly critical for advancing effective immunotherapeutic strategies in OC.

Nowadays, numerous studies on T cells have primarily focused on tumor-infiltrating CD8^+^ T cells due to their critical role in killing tumor cells. However, as is well known, CD8^+^ T cells do not function in isolation. Conventional CD4^+^ helper T (THC) cells also have a contributing role in anti-tumor immune response [12,13,14]. THCs promote CD8^+^ T cells priming through CD40L-CD40 interactions with dendritic cells (DCs) [14]. In addition to THC cells, CD4^+^ T follicular helper (TFH) cells have been recognized as crucial mediators that support B-cell activation and proliferation in human tumors [15]. In contrast, CD4^+^ regulatory T (Treg) cells promote tumor progression by suppressing tumor-specific immunity, including CD8^+^ T-cell activity [16]. Overall, tumor-infiltrating CD4^+^ T cells play an essential role in immunotherapy, comparable to that of CD8^+^ T cells. Nevertheless, the role of CD4^+^ T cells in the ovarian cancer (OC) tumor microenvironment remains significantly understudied. The emergence of single-cell RNA sequencing (scRNA-seq) has revolutionized the field by enabling the transcriptomic profiling of complex tissues at single-cell resolution. This technology not only reveals mutation-induced transcriptional heterogeneity and epigenetic modifications but also uncovers previously uncharacterized cellular biomarkers [17,18,19]. Recent advances in scRNA-seq technology, in combination with bulk RNA-seq data, now permit the characterization of tumor-infiltrating CD4^+^ T cells at the single-cell level.

**Figure 1:**
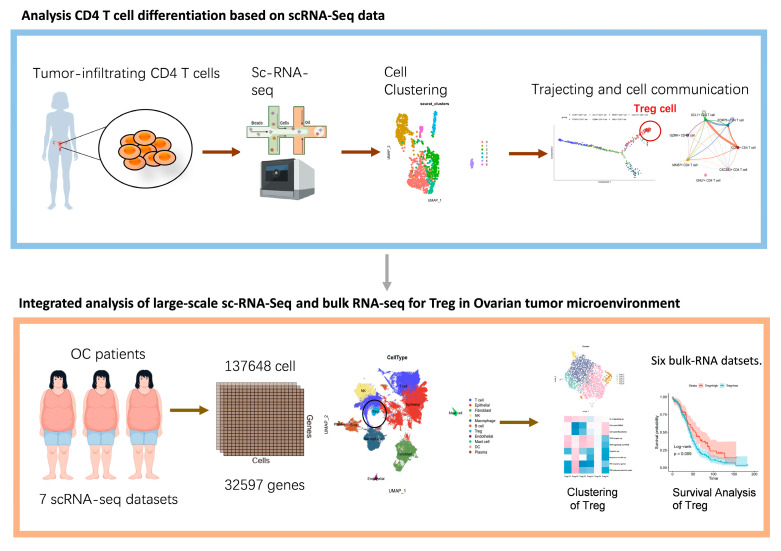
The workflow of the present study.

In this research, scRNA-seq was employed to delineate the subtypes and differentiation trajectories of tumor-infiltrating CD4^+^ T cells in ovarian cancer. Based on pseudotime analysis, the final states (FOXP3^+^ Treg cell and STMN1 proliferating T cell) of CD4^+^ T-cell differentiation were determined among seven functionally defined subsets (C1–C7). Subsequent analyses focused on the role and characteristics of Treg cells within the ovarian cancer tumor microenvironment (TME). To enable a comprehensive investigation, seven different datasets of OC from multicenter studies, called ovarian cancer scRNA-seq datasets (OCSCDs), were integrated in order to create abundant metadata for the in-depth analysis of Treg in immune microenvironment. This integrated dataset contained a total of 137,648 cells, including 4533 Treg cells. Using bulk RNA-seq data from The Cancer Genome Atlas (TCGA-OV), patients were grouped according to Treg scores, which were calculated by the Bayes Prism algorithm. The high Treg group and the low Treg group were compared in terms of survival, mutated genes, and immune subtypes. In addition to TCGA-OV, five independent bulk RNA-seq datasets based on different platforms were selected for survival analysis between the high and low Treg groups. Finally, we conducted experiments to validate the immunosuppressive function of Treg cells. The results confirm that Treg cells exert strong immunosuppressive activity within the ovarian cancer microenvironment. Figure 1 is the workflow of this research.

## 2. Materials and Methods

### 2.1. Data Acquisition and Handling

The scRNA-seq dataset of tumor-infiltrating CD4^+^ T cells in ovarian cancer (GSE156728) was obtained from the Gene Expression Omnibus (GEO) database. The tumor-infiltrating CD4^+^ T-cell samples were converted into a Seurat object using the “CreateSeuratObject” function from the Seurat R package [20] (v4.2.2). The comprehensive scRNA-seq dataset for OC was composed of multiple GEO datasets, including GSE181955, GSE165897, GSE158937, GSE147082, GSE130000, GSE118828, and GSE160754. To enable effective integration and comparison across datasets, dimensionality reduction and batch correction were performed using the Harmony method. After standard preprocessing with Seurat, the Harmony algorithm was applied to mitigate batch effects across different samples. Bulk RNA-seq datasets were obtained from GSE18520, GSE140082, GSE49997, GSE32063, and GSE23554, corresponding to the platforms GPL570, GPL14951, GPL2986, GPL6480, and GPL96, respectively.

### 2.2. Clustering of scRNA-Seq Data

We used Seurat’s FindIntegrationAnchors function to integrate the normalized scRNA-seq datasets of tumor-infiltrating CD4^+^ T cells. After identifying the top 2000 mainly using the SelectIntegrationFeatures method, we applied data scaling and principal component analysis (PCA) dimensionality reduction. Tumor-infiltrating CD4^+^ T cells were identified using the FindClusters and FindNeighbors (resolution = 0.5). To visualize the resulting cell clusters, we employed the Uniform Manifold Approximation and Projection (UMAP) algorithm. According to large-scale scRNA-seq datasets of OC, clustering followed a previously described protocol, with the resolution parameter set to 0.8.

### 2.3. Cell-Type Identification Analysis of Tumor-Infiltrating CD4^+^ T Cells

Subtype annotation of clustered tumor-infiltrating CD4^+^ T cells was performed based on T-cell identity markers. Marker genes for CD4^+^ T-cell subtypes were obtained by querying the CellMarker 2.0 Database. The main parameters of CD4^+^ T-cell data handling were set as follows: logfc.threshold = 0.25, *p* < 0.05, and min.pct = 0.25. Predicted cells from the OC tumor microenvironment were roughly annotated by SingleR in the first step. Then, in order to identify the feature of different cell clusters, differentially expressed genes (DEGs) were identified using the FindAllMarkers function. The same parameters (logfc.threshold = 0.25, *p* < 0.05, min.pct = 0.25) were applied to the analysis of the large-scale datasets.

### 2.4. Analysis of Cell Communication

The CellChat package [21] (v1.5.0) was used to infer cell–cell communication networks from scRNA-seq data by characterizing ligand–receptor–cofactor interactions. To investigate potential cross-talk involving tumor-infiltrating CD4^+^ T cells, CellChat was employed to model intercellular signaling networks. In terms of OCSCDs, R package CellChat was further used to analyze the communication between Treg cells and other cells.

### 2.5. Trajectory Analysis

Differentiation trajectories of tumor-infiltrating CD4^+^ T cells were reconstructed using Monocle’s pseudotime ordering algorithm [22]. The reduceDimension function was executed on selected genes with default settings (method = DDRTree). Developmental ordering was performed with orderCells (reverse = FALSE) on dimensionality-reduced gene expression. Afterwards, the plot_cell_trainjection function was used to visualize the differentiation trajectory, incorporating cell grouping, cell status, virtual time, and annotated cell subpopulations.

### 2.6. Gene Set Enrichment in Treg Cluster of OCSCDs

We performed Gene Set Enrichment Analysis (GSEA) to identify enriched functional pathways across transcriptional clusters. The competitive gene set enrichment method CAMERA was adapted and applied to perform gene set enrichment analysis [23]. The GSEA was implemented using the SingleSeqGset R package (https://github.com/arc85/singleseqgset, accessed on 17 July 2025).

### 2.7. Assessment of Treg Cells in OCSCDs

OCSCDs were deconvoluted using BayesPrism [24] (https://github.com/Danko-Lab/BayesPrism, accessed on 17 July 2025) to establish a subtype-resolved signature matrix for Treg cells. Based on this Treg-specific feature matrix, immune cell deconvolution was applied to the tumor gene expression profiles of 371 TCGA ovarian cancer (OC) patients, producing a matrix of regulatory T-cell (Treg) abundance scores. As the Treg score increases, the absolute proportion of Treg cells within tumor tissue also increases. Patients from TCGA OC were classified to low Treg and high Treg groups using the Wilcox test.

### 2.8. Differential Expression and Functional Enrichment Analysis in Low Treg Group and High Treg Group

Differential expression analysis of mRNA, phosphorylated proteins, and total protein expression profiles were performed using the “DESeq2” R package [25] (v3.19) between the low Treg group and high Treg group in OV TCGA-OV samples. Phosphorylation and protein expression data were obtained from cBio Cancer Genomics Portal (cBioPortal) (cBioPortal for Cancer Genomics). *p* < 0.05 and |log2 FC| ≥ 1 were selected as the thresholds for filtering differentially expressed genes and phosphorylation protein. Functional enrichment for Kyoto Encyclopedia of Genes and Genomes (KEGG) pathways and Gene Ontology (GO) terms were conducted using the clusterProfiler package (v4.16.0) [26].

### 2.9. Mutated Gene and Immune Subtype Analysis in Low Treg Group and High Treg Group

Mutated genes in the low Treg group and high Treg group were obtained from the cBioPortal database. A divisive hierarchical clustering algorithm [27] was used to construct sample dendrograms, with 1000 bootstrap replicates (80% patient subsampling per iteration) to assess cluster stability. The optimal number of clusters (ranging from 2 to 6) was determined by evaluating the consensus matrix stability and analyzing the characteristics of the cumulative distribution function (CDF) curve.

### 2.10. Immune Signature Analysis and Prediction of Treatment Sensitivity

Based on dozens of previously published gene signatures related to tumor mutation burden (TMB) and immune exclusion collected in the IOBR package (v2.0.0) [28], enrichment scores for each sample were calculated using the single-sample gene set enrichment analysis (ssGSEA) method. The scores of Microsatellite instability (MSI) were downloaded using the cBioPortalData R package (v2.21.5) to analyze MSI differences between two groups. Subclass mapping (Submap) and Tumor Immune Dysfunction and Exclusion (TIDE) algorithms were used to estimate the response to immunotherapy [29]. The Submap algorithm was applied using the GSE91061 immunotherapy dataset for mapping. The oncoPredict R package (https://github.com/maese005/oncoPredict, accessed on 17 July 2025) was utilized to estimate the IC50 values of drugs for each ovarian cancer sample, based on data from the Genomics of Drug Sensitivity in Cancer (https://www.cancerrxgene.org/, accessed on 17 July 2025).

### 2.11. Survival Analysis Related to Low Treg Group and High Treg Group

For validation, survival curves generated using the “survival” and “survminer” R packages to visualize the survival differences between the high Treg and low Treg groups.

### 2.12. Cell Culture and Sample Extraction

Human ovarian cancer cell lines (SKOV3) and human normal ovarian epithelial cells (IOSE80) were obtained from the American Type Culture Collection (ATCC) and cultured in DMEM supplemented with 10% fetal bovine serum (FBS) and 1% penicillin–streptomycin (Pen-Strep). Peripheral blood mononuclear cells (PBMCs) were isolated by IPHASE Biosciences (Suzhou, China). The sorted cells were resuspended in complete RPMI-1640 medium (supplemented with 10% FBS, 1% penicillin–streptomycin, and 2 mM L-glutamine) and seeded into anti-CD3 pre-coated 24-well plates. The culture medium contained anti-CD28 (1 μg/mL), recombinant human IL-2 (20 ng/ mL), and recombinant human TGF-β1 (5 ng/mL). After 3 days, cells were washed and resuspended in fresh complete RPMI-1640 medium supplemented with IL-2 (20 ng/mL) and maintained in culture until day 7. CD8^+^ T cells were also isolated from freshly prepared PBMCs of healthy donors using FACS. The cells were resuspended in complete RPMI-1640 medium and seeded into anti-CD3-coated 24-well plates, with culture medium containing anti-CD28 (1 μg/mL) and IL-2 (20 ng/mL). All cells were maintained at 37 °C in a 5% CO_2_ incubator.

### 2.13. RNA Extraction and RT-qPCR

Total RNA was isolated using TRIzol Reagent (Invitrogen, Carlsbad, CA, USA) following the manufacturer’s protocol. Complementary DNA (cDNA) was synthesized with M-MLV Reverse Transcriptase (Vazyme Biotech, Nanjing, China). Gene expression levels of FOXP3, TGF-β, and IL-10 were quantified by real-time PCR using SYBR Green Master Mix (Vazyme Biotech, Nanjing, China) on a 7500 Real-Time PCR System (Applied Biosystems, Foster City, CA, USA). Primers for FOXP3, TGF-β, and IL-10 were synthesized by Tsingke Biological Technology (Nanjing, China). GAPDH was used as the endogenous control for the normalization of mRNA relative expression levels, which were calculated using the 2^−ΔΔCt^ method.

### 2.14. CCK-8 and Transwell Assay

In vitro-activated CD8^+^ T cells and Tregs were co-cultured in 96-well plates at varying ratios (1:0, 1:1, 2:1), with CD8^+^ T cells seeded at 6 × 10^4^ cells/well. The corresponding Treg numbers were 0, 6 × 10^4^, and 3 × 10^4^ cells/well. Ovarian cancer cells were added to at a 1:20 ratio relative to CD8^+^ T cells (3 × 10^3^ cells/well). A control group containing only ovarian cancer cells (no lymphocytes) was included. After 48 h of tri-culture (ovarian cancer cells + CD8^+^ T cells +Tregs), wells were washed 2–3 times with PBS to remove non-adherent lymphocytes and necrotic tumor cells. CCK-8 reagent (Beyotime, Shanghai, China) was added to the remaining adherent cells. Following 2 h of incubation at 37 °C, absorbance at 450 nm was measured using a microplate reader. Treg-mediated suppression of CD8^+^ T-cell cytotoxicity against tumor cells was quantified by comparing absorbance values to lymphocyte-free control groups.

After 48 h of co-culture, cells were washed 2–3 times with PBS, trypsinized, and seeded into 6-well plates for subsequent experiments. For the migration assay, cells were plated in Transwell inserts at 2 × 10^4^ cells/well in serum-free medium. The lower chamber contained complete medium with 10% fetal bovine serum (FBS). After 72 h of culture (triplicate wells per group), inserts were fixed and stained with 0.1% crystal violet. Migrated cells were imaged and quantified using the Image J software (V1.8.0.112), with results expressed as mean ± standard deviation (SD) from three independent experiments.

## 3. Results

### 3.1. Characterization of Tumor-Infiltrating CD4^+^ T Cells in OC

**Figure 2:**
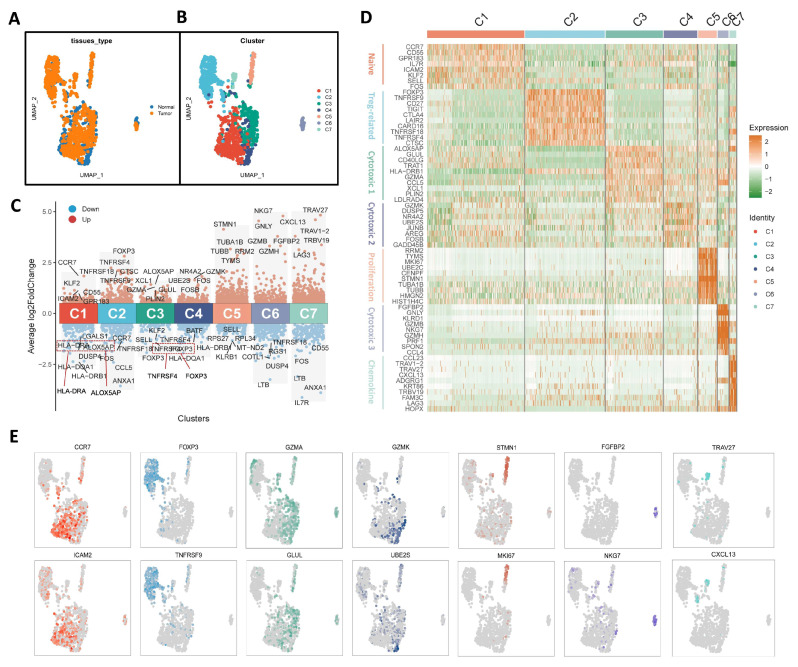
Characterization of tumor-infiltrating CD4^+^ T cells in ovarian cancer. (**A**) UMAP clustering of tumor-infiltrating CD4^+^ T cells based on different samples. (**B**) UMAP clustering of tumor-infiltrating CD4^+^ T cells based on cell types. (**C**) Volcanic diagram of CD4^+^ T cell subgroups with upregulated and downregulated genes. (**D**) Heatmap of top 10 characteristic genes in 7 clusters. (**E**) Scatter diagram of gene expression with different subpopulation characteristics.

We analyzed 1357 CD4^+^ T cells from tumor and adjacent tissues (Figure 2). After a series of processing steps, 1279 tumor-infiltrating CD4^+^ T cells were retained and subsequently segregated into two groups based on their tissue of origin. Blue represents adjacent cancer samples (n = 273), while orange represents tumor tissue (n = 1006) (Figure 2A). These cells are divided into seven major subgroups, designated as C1 through C7. Comparative analysis revealed that subgroups C2 and C5 were more prevalent in tumor samples, whereas subgroup C4 was more enriched in adjacent normal tissue (Figure 2B). Figure 2C displays the top five upregulated genes (red) and downregulated genes (blue) for each the C1 to C7 subgroups. Notably, expression of CCR7 is markedly elevated in the C1 subgroup. Based on fundamental immunological knowledge, CCR7 is a key marker gene for naïve T cells. Therefore, C1 was identified as the naïve CD4^+^ T-cell subset. FOXP3 is a characteristic gene of regulatory T cells, indicating that the C2 subgroup represents Treg cells. GZMA and GZMK are cytotoxic T cell-associated genes, suggesting that the C3 and C4 subgroups correspond to cytotoxic CD4^+^ T cells. STMN1 is a marker gene associated with T-cell proliferation, identifying the C5 subgroup as proliferative T cells. The characteristic expression genes of the C6 subgroup are NKG7 and GNLY, which are a special T-cell subcluster with both NK-cell receptor and T-cell receptors (TCRs) on their surface. These NKT-like cells produce high levels of cytokines and exhibit cytotoxic activity similar to NK cells; thus, the C6 subgroup was classified as a cytotoxic T-cell subtype. The C7 subgroup shows upregulated expression of CXCL13, suggesting it comprises a chemokine-associated T-cell population. According to previous reports, CXCL13 is also one of the genetic markers of follicular T-helper cells; so, the C7 subgroup may be follicular germinal center helper T cells. In terms of downregulating genes, the expression level of LGALS1 is relatively low in the C1 subgroup; ANXA1 is significant downregulated in the C2 and C7 subgroups. The expression levels of SELL are relatively low in both C3 and C5; LTB is downregulated in both C6 and C7. In addition, CCR7 and CCL5 have lower expression levels in the C2 subgroup, while IL7R is most significantly downregulated in the C7 subgroup. To provide a more intuitive view of gene expression across subpopulations, a selection of annotated genes in Figure 2C was used to generate a subpopulation scatter plot, as shown in Figure 2E.

### 3.2. Trajectory of Tumor-Infiltrating CD4^+^ T Cells

**Figure 3:**
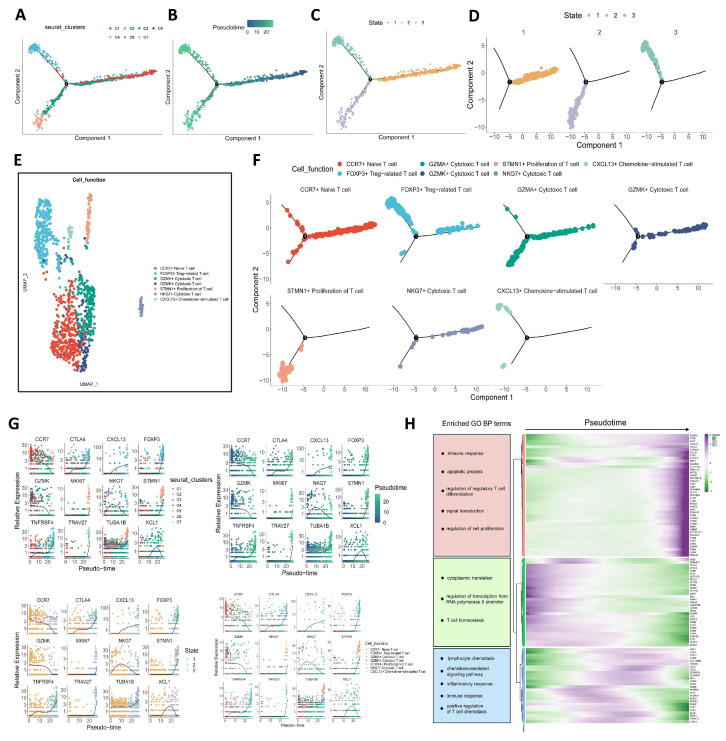
Pseudotime trajectories analysis of tumor-infiltrating CD4^+^ T cells in ovarian cancer. (**A**) Trajectory of CD4^+^ T cell differentiation classified by cell subpopulation. (**B**) Trajectory of CD4^+^ T cells classified by pseudotime. (**C**) Trajectory of CD4^+^ T cells classified by cell state. (**D**) Three cell states of CD4^+^ T cells. (**E**) The UMAP dimensionality reduction graph of the distinct clusters, which were annotated by cellular function. (**F**) Trajectory of CD4^+^ T cells classified by clusters, which were annotated by cellular function. (**G**) Changes in expression levels of selected genes during cell differentiation in different classifications. (**H**) Heatmap of corresponding genes and pathways in pseudotime trajectories analysis.

We performed pseudotime trajectory analysis of OC tumor-infiltrating CD4^+^ T cells using Monocle 2. As shown in Figure 3A, different colors represent different cell clusters, and the differentiation states of the two branch terminals are the C2 and C5 subgroups. Figure 3B illustrates the trajectory of cell differentiation along a virtual time axis, where the color gradient of the cell points transitions from dark to light, indicating progression through pseudotime. During the process of cell differentiation, tumor-infiltrating CD4^+^ T cells were roughly divided into three states with the intermediate node as the critical point (Figure 3C,D). In Figure 3E,F, CCR7^+^ naïve-like cells are predominantly concentrated in state 1. Although a small number of Treg cells also appear in state 1, the majority are primarily localized in state 3, particularly at the terminal end. Based on the trajectory analysis of CD4^+^ T cells, the differentiation path bifurcates into two major branches: one branch culminates in Tregs, while the other leads to a population of T cells with high proliferative capacity. Figure 3G reflect the changes in expression levels of genes selected based on different classifications during cell differentiation. Among the selected genes, CTLA4, FOXP3, MK167, STMN1, TRAV27, TNFRSF4, and TUBA1B showed increased expression levels during the differentiation process; then, the rest are all decreasing. Based on the selected characteristic genes, these genes were divided into three modules by pseudotime analysis, and the quasi-temporal direction was from left to right, and the color gradually changed from green to purple, which could reflect the changes in gene expression during cell differentiation. At the same time, the enrichment analysis of the gene sets of three different modules was also carried out, and the main enrichment pathways are marked in Figure 3H.

### 3.3. Cell–Cell Communication Analysis

**Figure 4:**
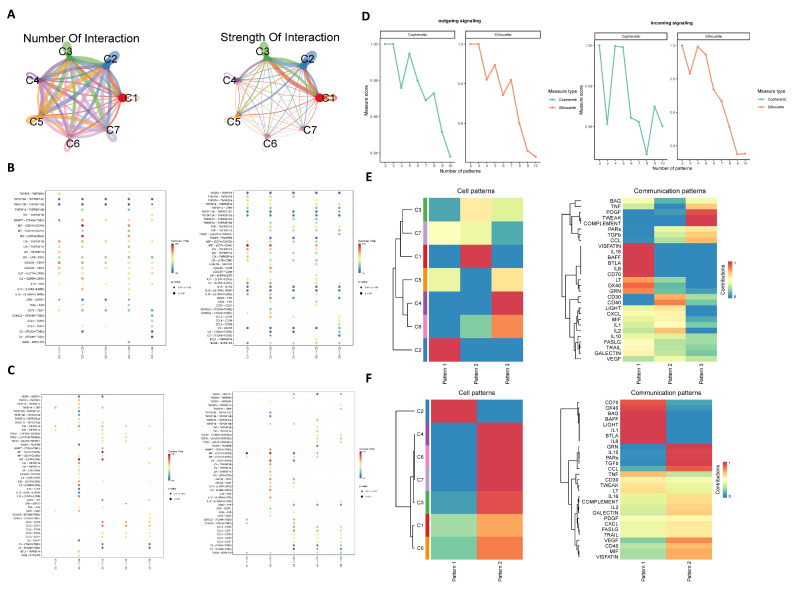
Cell–cell communication analysis of tumor-infiltrating CD4^+^ T cells in ovarian cancer. (**A**) The quantity and intensity of cell communication between different subgroups. (**B**) Ligand–receptor interactions between the Treg-cell subpopulation (C2) and other cell subpopulations. (**C**) Ligand–receptor interactions between proliferated T-cell subpopulation (C5) and other cell subpopulations. (**D**) Transmission signal patterns analysis of ligand–receptor cell interactions. (**E**) Heatmap of incoming communication modes for various subgroups and related pathways. (**F**) Heatmap of outcoming communication modes for various subgroups and related pathways.

Frequency (left) and strength (right) of cell–cell communication among subgroups C1~C7 were visualized by using the netVisual_circle function (Figure 4A). Strong correlations were observed among C1, C2, and C3, where C1 represents the initial T-cell subset, C2 is Treg cell subset, and C3 is cytotoxic T-cell subset. According to the results of the pseudotime analysis, the final differentiation state of the cell is FOXP3^+^Treg cells (C2) and STMN1 proliferating T cells (C5). C2 and C5 act as ligands and receptors, respectively. As shown in Figure 4B,C, the MIF signaling pathway mediated correlations between C2/C5 and other clusters, predominantly through MIF-CD74^+^CXCR4 and MIF-CD74^+^CD44 receptor complexes. We further investigated the communication patterns between cells using the NMF algorithm. In Figure 4D, when the number of incoming patterns is set to 2, both the Cophenetic and Silhouette values drop sharply, suggesting an optimal division into two cell modules (Figure 4E). Similarly, when the number of outgoing patterns is set to 3, a similar decline is observed, indicating a division into three cell modules (Figure 4F).

### 3.4. Characterization and Cell–Cell Communication of Treg Cells in OCSCDs

**Figure 5:**
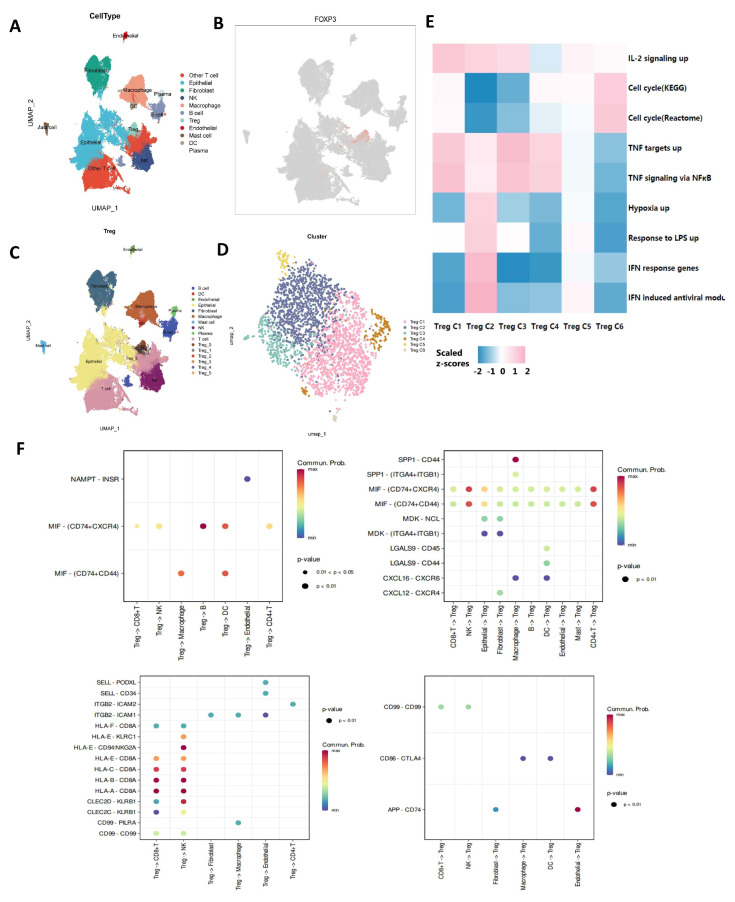
Characterization and cell–cell communication of Treg cells in ovarian cancer tumor infiltration single-cell datasets (OCSCDs). (**A**) Distinct cell types in OCSCDs. (**B**) FOXP3 expression level in each cluster. (**C**) Subtypes of Treg cells in OCSCDs. (**D**) Subtypes of Treg cells. (**E**) Heatmap of pathway related to different subtypes of Treg cells. (**F**) Cell–cell communication of Treg cells.

The OCSCD dataset comprises seven datasets totaling 137648 single cells. Samples within OCSCDs were visualized using UMAP dimensionality reduction (Figure S1). UMAP projection of initial cluster assignments in OCSCDs (Figure S2). Expression patterns of signature genes in OCSCD subpopulations provide basis for cell annotation (Figure S3). As shown in Figure 5A, these single cells are divided into 11 clusters: Macrophage, Treg, B cell, DC, NK, Epithelial, Fibroblast, Endothelial, Plasma, Mast cell, and Other T cell. The most typical characteristic gene for Tregs is FOXP3, highlighted in red in Figure 5B. In order to further explore different subtype of Treg cells intensively, Treg cells in the tumor microenvironment of OC were classified into six clusters, named Treg C1, Treg C2, Treg C3, Treg C4, Treg C5, and Treg C6 (Figure 5C,D). Then cluster-associated biological mechanisms were investigated using gene set enrichment analysis (GSEA) (Figure 5E). Notably, Treg C2 exhibited enrichment for both IFN-α response gene sets and broad-spectrum IFN-induced antiviral modules, whereas enrichment of tumor necrosis factor receptor TNFR pathway signatures was observed in Treg C1, Treg C3, and Treg C4. IFN response and TNFR signaling modules were differentially enriched in distinct differentiation states, suggesting cellular responses are tailored to specific signals. CellChat analysis was used to decode ligand–receptor-mediated communication between Tregs and other cell types (Figure 5F). Treg-B cell interactions were primarily driven by MIF signaling via MIF-CD74^+^CXCR4. SPP1 secretion by macrophages binding CD44 on Tregs constituted the primary intercellular communication mechanism. Additionally, APP-CD74 was the dominant ligand–receptor pair in endothelial Treg signaling.

### 3.5. Differential and Enrichment Analysis Related to Treg Cells

**Figure 6:**
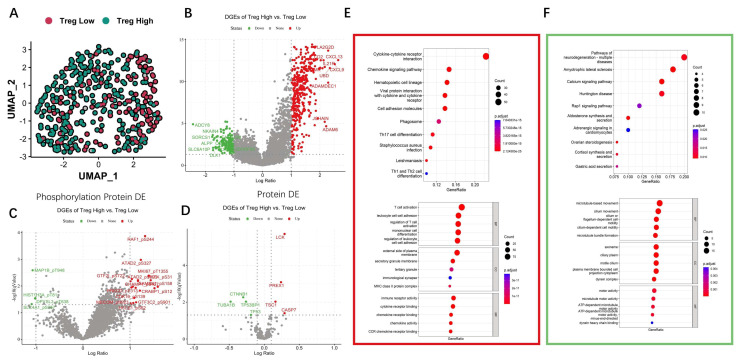
Differential analysis and enrichment analysis of high Treg group and low Treg group in TCGA-OV dataset. (**A**) Scatter plot of OV samples based on high Treg group and low Treg group. (**B**) Volcano plot of differentially expressed genes. (**C**) Volcano plot of differentially expressed phosphorylation proteins. (**D**) Volcano plot of differentially expressed proteins. (**E**) KEGG and GO enrichment analyses of overexpressed genes between two groups. (**F**) KEGG and GO enrichment analyses of downregulated genes between two groups.

According to the research above, the scores of Treg cell immune infiltration were calculated by the Bayes Prism algorithm. Based on Treg levels, TCGA-OV samples were categorized into high Treg and low Treg groups. Figure 6A indicates that the distribution of high Treg group (green) and high Treg group (red) in TCGA-OV samples. To investigate the molecular differences between these two groups, 493 genes were upregulated and 256 genes were downregulated in TCGA-OV samples (Figure 6B, *p* < 0.05 and |log2 FC| ≥ 1). A total of 19 phosphorylation proteins were upregulated and 4 phosphorylation proteins were downregulated in TCGA-OV samples (Figure 6C, *p* < 0.05 and |log2 FC| ≥ 1). Four proteins were upregulated and four proteins were downregulated in TCGA-OV samples (Figure 6D, *p* < 0.05 and |log2 FC| ≥ 0.1).

GO and KEGG functional enrichment analyses were performed based on upregulated and downregulated genes (Figure 6E,F). For upregulated genes, the most significantly enriched KEGG pathway was cytokine–cytokine receptor interaction. The GO enrichment analysis demonstrated a significant enrichment of upregulated genes in T-cell activation, leukocyte cell–cell adhesion, regulation of T cells, mononuclear cell differentiation, and regulation of leukocyte cell–cell adhesion. Cellular components (CCs) were specifically related to external side of plasma and secretory granule membrane. For molecular function (MF), the upregulated genes were primarily associated with immune receptor activity, cytokine receptor binding, chemokine receptor binding, chemokine activity, and CCR chemokine receptor binding. With regard to the downregulated genes, KEGG enrichment analysis revealed significant enrichment in three pathways: pathways of neurodegeneration—multiple diseases, amyotrophic lateral sclerosis, and calcium signaling pathway. GO enrichment analysis revealed significant enrichment of biological processes (BPs) related to microtubule-based movement, cilium movement, cilium or flagellum-dependent cell motility, cilium-dependent cell motility, and microtubule bundle formation. CCs were mainly associated with axoneme, ciliary plasm, motile cilium, and plasma membrane-bound cell projection complex. MF was mainly related to motor activity, microtubule motor activity, and ATP-dependent microtubules.

### 3.6. Survival Analysis, Mutated Gene, and Immune Subtype Analysis

**Figure 7:**
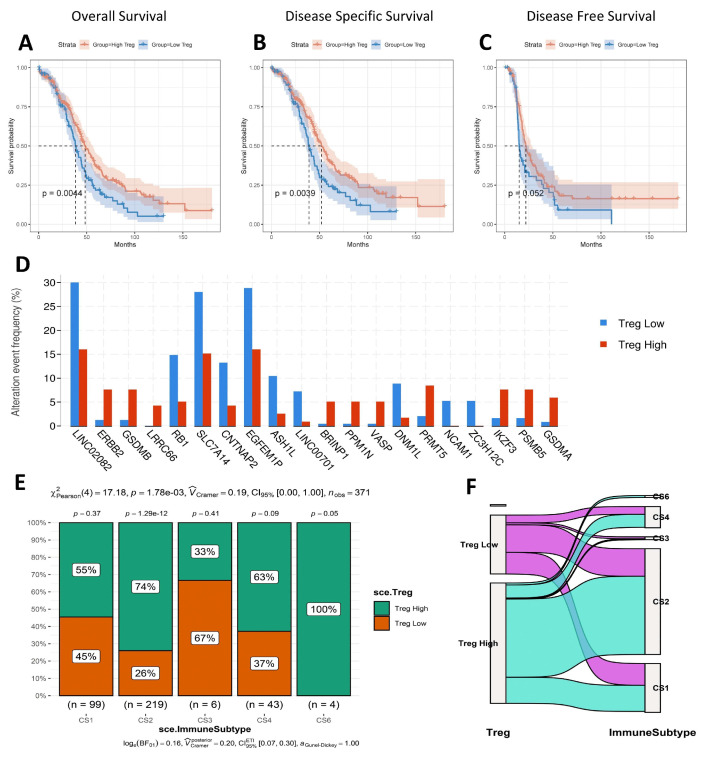
Survival analysis, mutated gene, and immune subtype analysis of high Treg group and low Treg group in TCGA-OV dataset. (**A**) Kaplan–Meier plot of overall survival (log-rank test). (**B**) Kaplan–Meier plot of disease specific survival (log-rank test). (**C**) Kaplan–Meier plot of disease-free survival (log-rank test). (**D**) Alteration event frequency of genes in two groups. (**E**) Comparison of immune subtypes based on two groups obtained from TCGA-OV by using ImmuneSubtypeClassifier. (**F**) River plot of grouped immune subtypes.

Using Bayes Prism-derived Treg scores, we investigated the association between tumor microenvironment Treg levels and ovarian cancer prognosis. Kaplan–Meier survival curves demonstrated significantly worse overall survival (OS), disease specific survival (DSS), and disease-free survival (DFS) in patients with high Treg scores (Figure 7A–C). Then, we also analyzed the alteration event frequency of top 20 genes in the high Treg group and low Treg group (Figure 7D). The results indicate that LINC02082, RB1, SLC7A14, CNTNAP2, EGFEM1P, ASH1L, LINC00701, DNM1L, NCAM1, and ZC3H12C were obviously expressed in the low Treg group. Genes of high mutation in the high Treg group were ERBB2, GSDMB, LRRC66, BRINP1, PPM1N, VASP, PRMT5, IKZF3, PSMB5, and GSDMA. The immune subtypes of TCGA-OC were accessed by ImmuneSubtypeClassifier. The C2 (CS2) immune subtype had the highest overall representation (n = 219), with 74% of CS2 cases belonging to the high Treg group and 26% to the low Treg group (Figure 7E,F).

### 3.7. Pathway Activity and TF Activity Analysis

**Figure 8:**
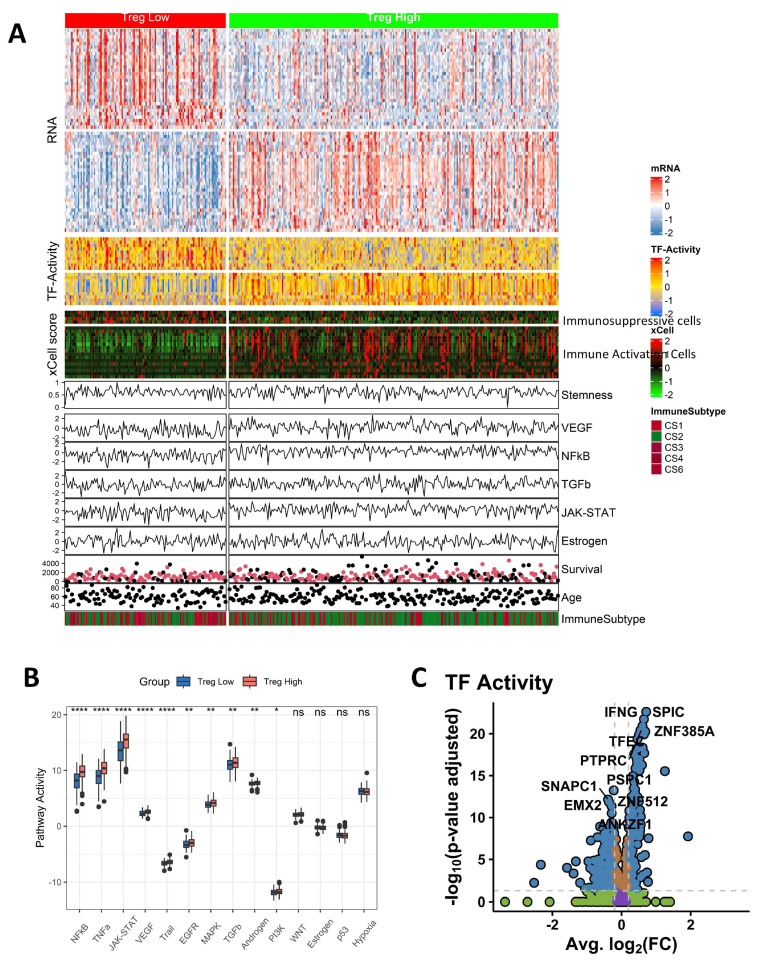
(**A**) Heatmap of high Treg group and low Treg group based on different indexes, including expression level of mRNA, TF-activity, xCell score, Stemness, pathway activity, survival, age, and immune subtype. (**B**) Comparison of pathway activity in the high Treg group and low Treg group. (**C**) Volcano plot of TF in two groups (* *p* < 0.05, ** *p* < 0.01, **** *p* < 0.0001).

To intuitively compare the high Treg group and low Treg group intuitively, Figure 8A demonstrates the expression level of mRNA, TF-activity, xCell score, Stemness, pathway activity, survival, age, and immune subtype between the high Treg group and low Treg group. We focused primarily on the analysis of pathway activity and TF activity (Figure 8B,C). Pathway analysis revealed that the high Treg group was enriched in the NFkB, YNFa, and JAK-STAT pathways compared to the low Treg group. In Figure 8C, the top ten transcription factors with elevated activity in the high Treg group were identified as IFNG, SPIC, ZNF385A, TFEC, PTPRC, PSPC1, SNAPC1, EMX2, ZNF512, and ANKZF1.

### 3.8. The Immune Landscape of Different Groups

**Figure 9:**
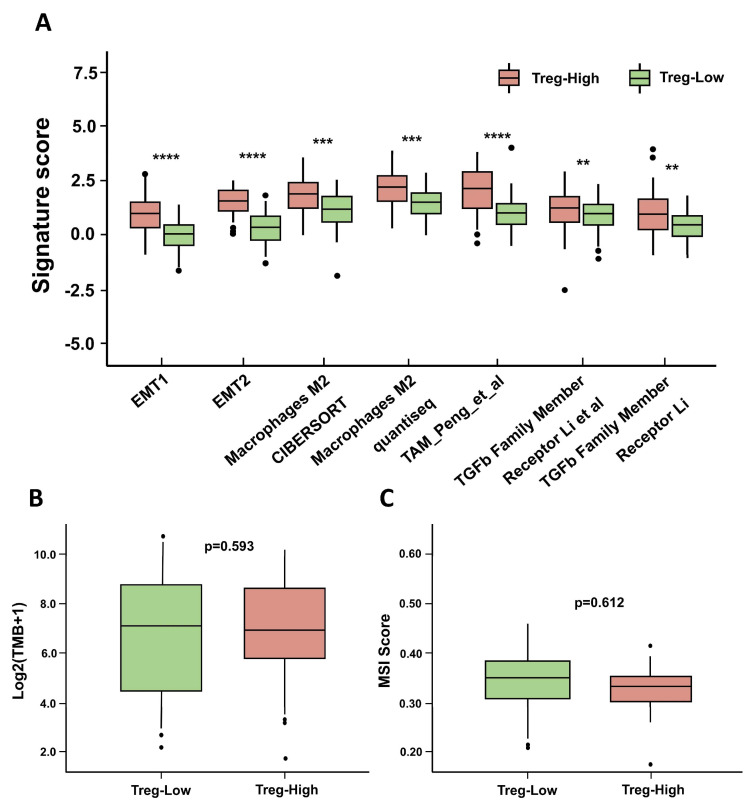
The immune landscape of different groups. (**A**) Immune exclusion. (**B**) TMB. (**C**) MSI. (** *p* < 0.01, *** *p* < 0.001, **** *p* < 0.0001).

Using various immune-related gene signatures collected from the IOBR package (v2.0.0), we conducted differential analyses of immune exclusion and tumor mutation burden (TMB). The results reveal that immune exclusion-associated pathways and cell types—such as EMT, TGF-β signaling, and M2 macrophages—were significantly enriched in the high Treg group (Figure 9A). This indicates that patients in the high Treg group are more likely to exhibit a “cold tumor” phenotype. Although tumors with high TMB typically generate more neoantigens and stimulate stronger T cell-mediated immune responses—resulting in improved responses to immune checkpoint inhibitors (ICIs)—no significant difference in TMB distribution was observed between the high Treg and low Treg groups (Figure 9B). These findings suggest that excessive infiltration of immunosuppressive cells and impaired T-cell activation may underlie the immunologically “cold” tumor microenvironment observed in the high Treg group. As shown in Figure 9C, the MSI values in both the high Treg and low Treg groups were below 0.4, with no significant differences between them, indicating that both groups were in a microsatellite stable (MSS) state. This finding suggests that no substantial length alterations were detected at the examined microsatellite loci, implying intact DNA mismatch repair (MMR) function and relative stability of microsatellite regions in the genome.

### 3.9. Immunotherapy and Drug Sensitivity Prediction

**Figure 10:**
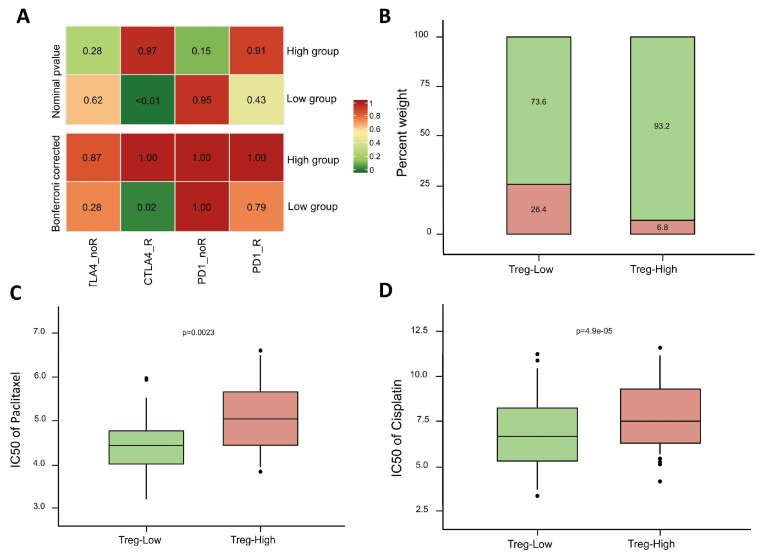
Response to immunotherapy and drugs in different groups. (**A**) Significance heatmap of responses to different immune checkpoints based on the SubMap algorithm. (**B**) Immunotherapy responses based on the TIDE algorithm. (**C**) IC50 of paclitaxel. (**D**) IC50 of cisplatin.

The SubMap algorithm indicated that the low Treg group exhibited a better response to CTLA-4 therapy, while no significant difference was observed between groups in response to PD-1 therapy (Figure 10A). Additionally, the analysis using the TIDE algorithm revealed that a higher proportion of patients in the low Treg group were predicted to respond to immunotherapy (26.4%) compared to only 6.8% in the high Treg group (Figure 10B). Drug sensitivity to cisplatin and paclitaxel was also evaluated across different groups. Sensitivity was assessed based on the value of IC50, with a higher IC50 indicating greater drug resistance. The results demonstrate that the low Treg group was more sensitive to both paclitaxel and cisplatin than the high Treg group (Figure 10C,D).

### 3.10. Survival Analysis Related to Treg Cells Based on Different Bulk RNA-Seq Datasets

**Figure 11:**
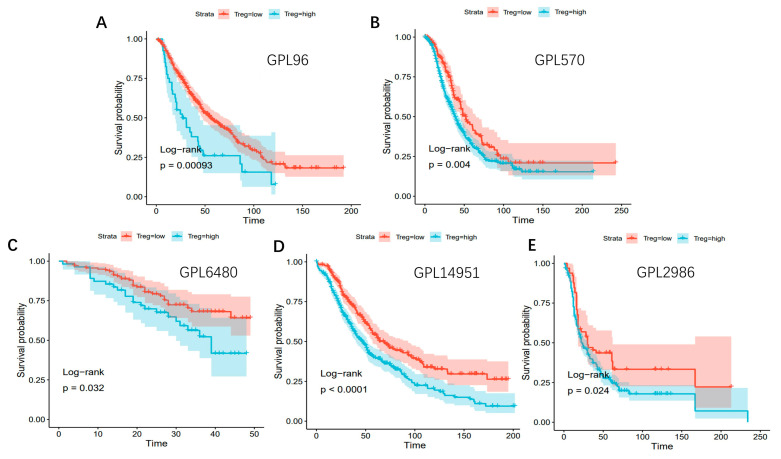
Survival analysis of high Treg group and low Treg group in different datasets based on different platforms. (**A**) GPL96. (**B**) GPL570. (**C**) GPL6480. (**D**) GPL14951. (**E**) GPL2986.

Bulk RNA-seq datasets, with their robust statistical power from large cohorts and integrated clinical annotations, serve as a valuable resource for exploring clinical relevance of Tregs in cancer progression and treatment response. Bayes Prism enables the estimation of cell-type composition and gene expression in tumors depend on count matrices from bulk RNA-seq. Using the TCGA dataset, the proportion of tumor-infiltrating regulatory T cells (Tregs) was quantified in OCSCDs via the Bayes Prism algorithm. Survival analyses across different bulk RNA-seq datasets from five platforms (GPL96, GPL570, GPL6480, GPL14951, and GPL2986) consistently showed that higher Treg infiltration was associated with poorer prognosis (Figure 11A–E). These findings highlight a significantly worse survival outcome for patients in the high Treg group compared to those in the low Treg group.

### 3.11. Experimental Validation

**Figure 12:**
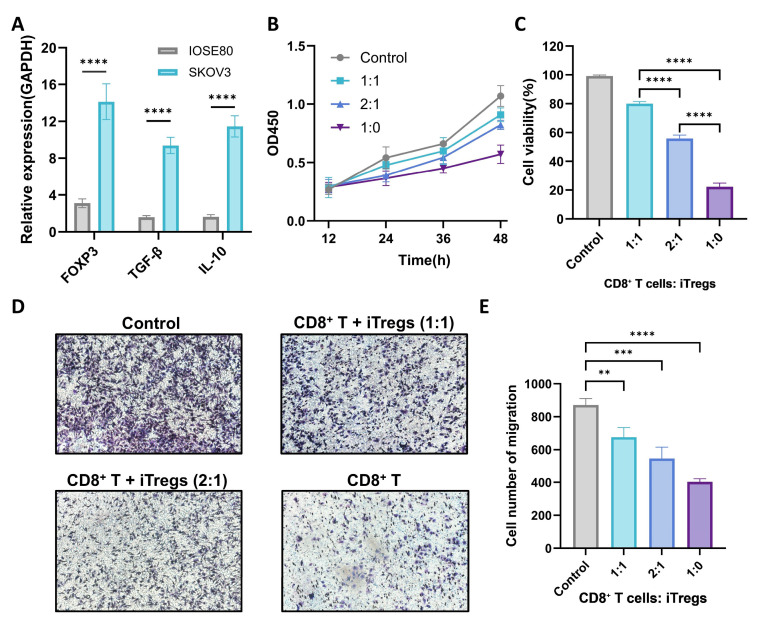
In vitro Treg suppression assay. (**A**) qPCR analysis of FOXP3, TGF-β, and IL-10 expression in Tregs after 48 h treatment with OC cell-conditioned supernatant versus control supernatant. Data normalized to GAPDH (n = 3 biological replicates). (**B**) Variation in OD450 values across different time points and cell ratios. (**C**) CD8^+^ T-cell cytotoxicity against ovarian cancer cells (SKOV3) co-cultured with iTregs at varying ratios (48h). (**D**) Cell migration of ovarian cancer cells (SKOV3) was assessed using Transwell assays. (**E**) Cell number of migration at different ratios. Data are presented as mean ± SD. Statistical significance was determined by a one-way ANOVA with Tukey’s post hoc test (** *p* < 0.01, *** *p* < 0.001, **** *p* < 0.0001).

Tregs secrete multiple immunosuppressive cytokines, including TGF-β and IL-10, which act through diverse signaling pathways to attenuate antitumor immune responses, drive the functional inactivation of effector immune cells and induce progressive exhaustion of activated CD8^+^ T cells [30]. In this study, iTregs were co-cultured for 48 h with conditioned media from either SKOV3 or IOSE80 cells. Total RNA was then extracted from the iTregs, and qPCR was performed to quantify the expression levels of FOXP3, TGF-β, and IL-10 in both groups. iTregs exposed to SKOV3-conditioned media exhibited significantly elevated FOXP3, TGF-β, and IL-10 expression (Figure 12A). These results suggest that soluble factors within the tumor microenvironment sustain high FOXP3 expression in Tregs, enhance their secretion of immunosuppressive cytokines, and thereby potentiate their immunosuppressive and metabolic regulatory capacities, ultimately further impairing antitumor immunity.

To further assess the inhibitory effect of iTregs on CD8^+^ T cell-mediated cytotoxicity against ovarian cancer cells, in vitro-activated CD8^+^ T cells were co-cultured in 96-well plates with varying ratios of iTregs (1:0, 1:1, and 2:1). The tri-culture system consisted of ovarian cancer cells + CD8^+^ T cells ± Tregs. After 12, 24, 36, and 48 h, CCK-8 reagent was added and incubated at 37 °C for 2 h, followed by measurement of absorbance at 450 nm (OD450) to determine the number of viable adherent tumor cells. As shown in Figure 12B, higher OD450 values indicated stronger suppression of CD8^+^ T-cell cytotoxicity by iTregs and increased tumor cell survival. Figure 12C presents the relative tumor cell viability after 48 h of co-culture. The results demonstrate a progressive decline in the cytotoxic activity of CD8^+^ T cells toward ovarian cancer cells as the proportion of Tregs increased.

To validate these findings, a Transwell migration assay was conducted. SKOV3 cells displayed high intrinsic migratory capacity, which was further enhanced in the presence of higher Treg-to-CD8^+^ T-cell ratios (Figure 12D,E). Collectively, these data indicate that tumor-infiltrating Tregs in ovarian cancer effectively suppress CD8^+^ T cell-mediated antitumor activity and facilitate immune evasion.

## 4. Discussion

Ovarian cancer is a global health challenge that is typically diagnosed at an advanced stage and currently lacks effective screening methods. The advent of scRNA-seq has rapidly advanced ovarian cancer research, enabling the exploration of tumor development, heterogeneity, and microenvironment dynamics [31]. Validating scRNA-seq findings with bulk RNA-seq data enhances the robustness and reliability of the results. In this study, we leveraged scRNA-seq to dissect the cellular diversity of gene expression in tumor-infiltrating CD4^+^ T cells, aiming to inform precision medicine approaches for ovarian cancer treatment. CD4^+^ T cells, particularly regulatory T cells (Tregs), can suppress CD8^+^ T-cell proliferation and cytotoxicity through inhibitory cytokines, IL-2 competition, and direct cell–cell contact, contributing to an immunosuppressive tumor microenvironment. Consistently, our study experimentally validated that Tregs inhibit CD8^+^ T-cell activity in ovarian cancer, and the observed differentiation trajectories suggest that the balance between proliferative and regulatory CD4^+^ T-cell states may modulate CD8^+^ T-cell function. These findings highlight the critical role of Treg-CD8^+^ T-cell interactions in anti-tumor immunity and support the potential of targeting Tregs to enhance CD8^+^ T cell-mediated responses, in line with previous reports in other cancer types.

Firstly, we analyzed the subtypes and differentiation of tumor-infiltrating CD4^+^ T cells in OC. Seven clusters (C1–C7) were classified by cell function and the differentiation trajectory was determined to two branches: FOXP3^+^ Treg cells (C2) and STMN1-proliferating T cells (C5). According to the results of cell–cell communication analysis among C2, C5, and other clusters, terminal states of tumor-infiltrating CD4^+^ T cells were specifically enriched in the MIF signal pathway (MIF-CD74^+^CD44, MIF-CD74^+^CXCR4). MIF modulates T-cell behavior via receptor interactions involving CD74 and co-receptors such as CD44 and CXCR2/4/7, leading to the downstream activation of chemotactic and survival signals. The MIF–CD74^+^CXCR4 axis, in particular, appears to drive inflammation-induced malignant transformation and cancer metastasis. FOXP3^+^ Treg cells are abundant at the differentiation terminal states of tumor-infiltrating CD4^+^ T cells. This means that FOXP3^+^ Treg cells maybe play a crucial in the tumor progression of OC. Following the results of previous study, patients with enriched CD137 high Treg populations exhibited significantly reduced overall survival [32]. TNFR superfamily receptor expression on Treg cells signals an effector state transition, aligning with their functional reprogramming [33]. To further investigate the role of Treg cells in the ovarian cancer tumor microenvironment, we constructed a large-scale integrated dataset (OCSCDs) from multiple scRNA-seq datasets. Treg cells within this dataset were grouped into six clusters (TregC1–Treg C6). According to the results of enrichment analysis, early-differentiated Treg cells exhibited reciprocal expression of IFNα signaling and general IFN response genes, contrasting with late-differentiation dominance of TNF receptor superfamily pathways. Building on IFN-γ-induced Treg fragility [34], it seems that early-activated Tregs with IFN-γ therapy versus chronically activated Tregs via TNFR signaling blockade.

In addition, we integrated bulk RNA-seq data from TCGA-OV with the OCSCD dataset for further analysis. The Bayes Prism algorithm was calculated to obtain the score of Treg-cell immune infiltration based on a deconvolution approach. The high Treg group and low Treg group were classified according to the aforementioned scores. Then, we conducted a series of comparisons between the two groups. Protein LCK (lymphocyte protein tyrosine kinase) was highly expressed based on the Treg group and low Treg group. During TCR triggering, LCK phosphorylates ITAM tyrosine residues on the cytoplasmic tails of CD3ζ, CD3ε, and CD3δ chains to initiate signal transduction [35,36]. A previous study showed that the pharmacological blockade of LCK/Fyn kinases augmented iTreg differentiation (from iTreg to similar tTreg) through activation of the AKT/mTOR signaling cascade [37]. Unlike the stable, immunosuppressive tTregs, iTregs are characterized by instability and susceptibility to differentiation into inflammatory T-cell lineages [38]. Therefore, the observed upregulation of LCK suggests a higher proportion of unstable Treg subpopulations in the high Treg group. Moreover, analysis of gene expression revealed that NCAM1 and ZC3H12C were significantly overexpressed in the high Treg group. Both of these genes may be immunosuppressive factors, which are related to the immune infiltration level of Treg cells. In terms of the result of survival analysis based on different bulk RNA-seq datasets, the higher Treg cell infiltration observed in high Treg patients compared to low Treg patients implies enhanced immunosuppressive potential. These findings underscore the importance of targeting Treg-mediated immunosuppression as a critical therapeutic strategy in the context of ovarian cancer immunotherapy.

## 5. Conclusions

In summary, this study delineated the terminal differentiation trajectories of tumor-infiltrating CD4^+^ T cells within the ovarian cancer microenvironment, identifying CD4^+^ regulatory T cells (Tregs) as the predominant subtype. By integrating single-cell RNA-seq data from seven multicenter ovarian cancer studies, we generated a comprehensive metadata resource to enable the in-depth characterization of Tregs in the tumor context. Patients were stratified into high Treg and low Treg groups based on infiltration scores derived from bulk TCGA RNA-seq data using the Bayes Prism algorithm. Furthermore, we presented survival analyses of bulk RNA-seq data from five other distinct platforms. Overall survival was significantly worse in the high Treg group compared to the low Treg group. This integrative, multi-modal RNA-seq analysis provides valuable insights that may inform the development of precision immunotherapy strategies for ovarian cancer. Finally, experimental validation further demonstrate that tumor-infiltrating Tregs suppress CD8^+^ T cell-mediated tumoricidal activity and promote immune evasion in ovarian cancer.

## Data Availability

All data generated in this study are publicly accessible via the associated web servers. These resources are freely available to the scientific community for non-commercial research use, with all participant confidentiality safeguards maintained. Subject to ethics approvals, Supplementary Materials may be requested from the corresponding author.

## Supplementary Materials

The following supporting information can be downloaded at: https://www.mdpi.com/article/10.3390/biom15091241/s1, Figure S1: CD4+T Cell differentiation trajectory analyzed using the Slingshot algorithm.; Figure S2: The UMAP dimensionality reduction graph of the different samples in OCSCDs; Figure S3: The UMAP dimensionality reduction graph of the primitive clusters in OCSCDs; Figure S4: The expression levels of marker genes in different clusters of OCSCDs; Table S1: The primer sequence used in this study.
