# Supplementary material for: Transcriptome Analysis Unravels CD4+ T-Cell and Treg-Cell Differentiation in Ovarian Cancer
Source: Biomolecules. 2025 Aug 27;15(9):1241. doi: 10.3390/biom15091241 (PMC12467124; doi:10.3390/biom15091241)
Supplement: Supplementary file 1 [file biomolecules-15-01241-s001.zip › biomolecules-3752258-SI.pdf]

Figure S1

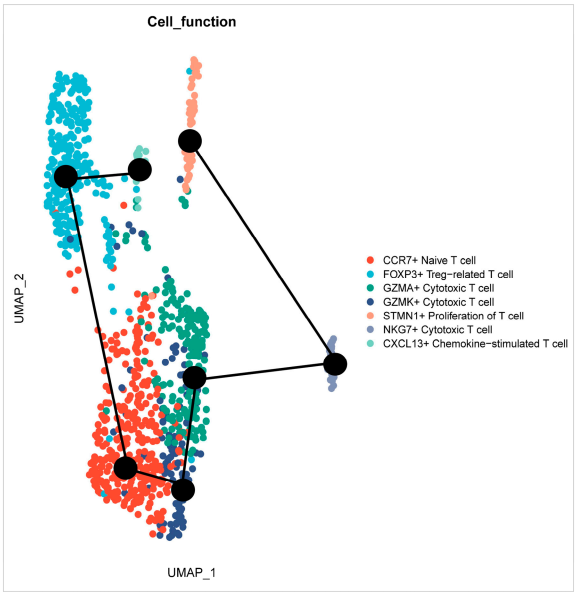

Figure S1 CD4+T Cell differentiation trajectory analyzed using the Slingshot algorithm.

Figure S2

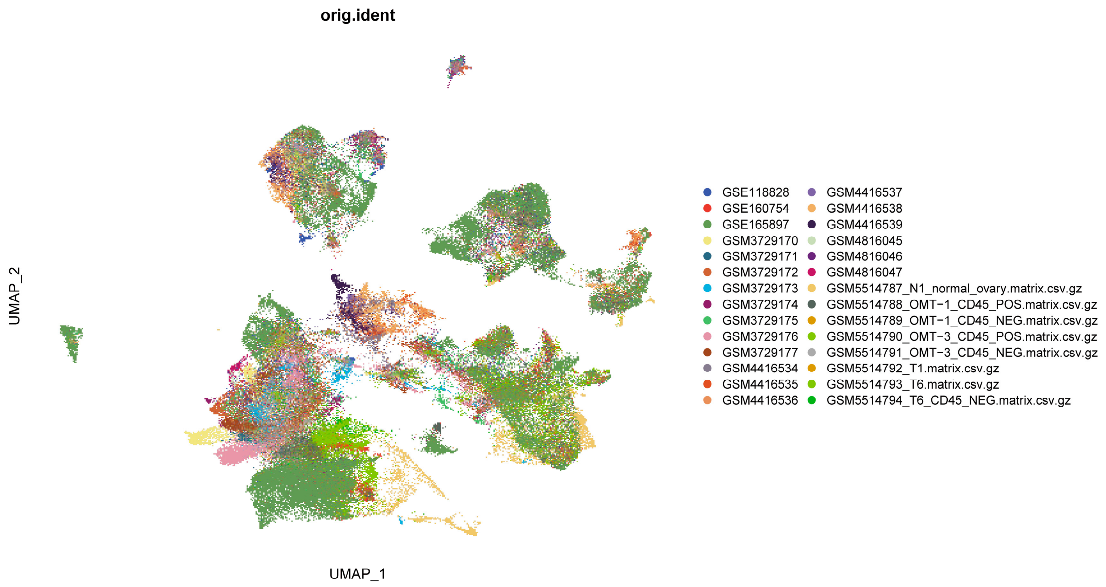

Figure S2 The UMAP dimensionality reduction graph of the different samples in OCSCDs.

Figure S3

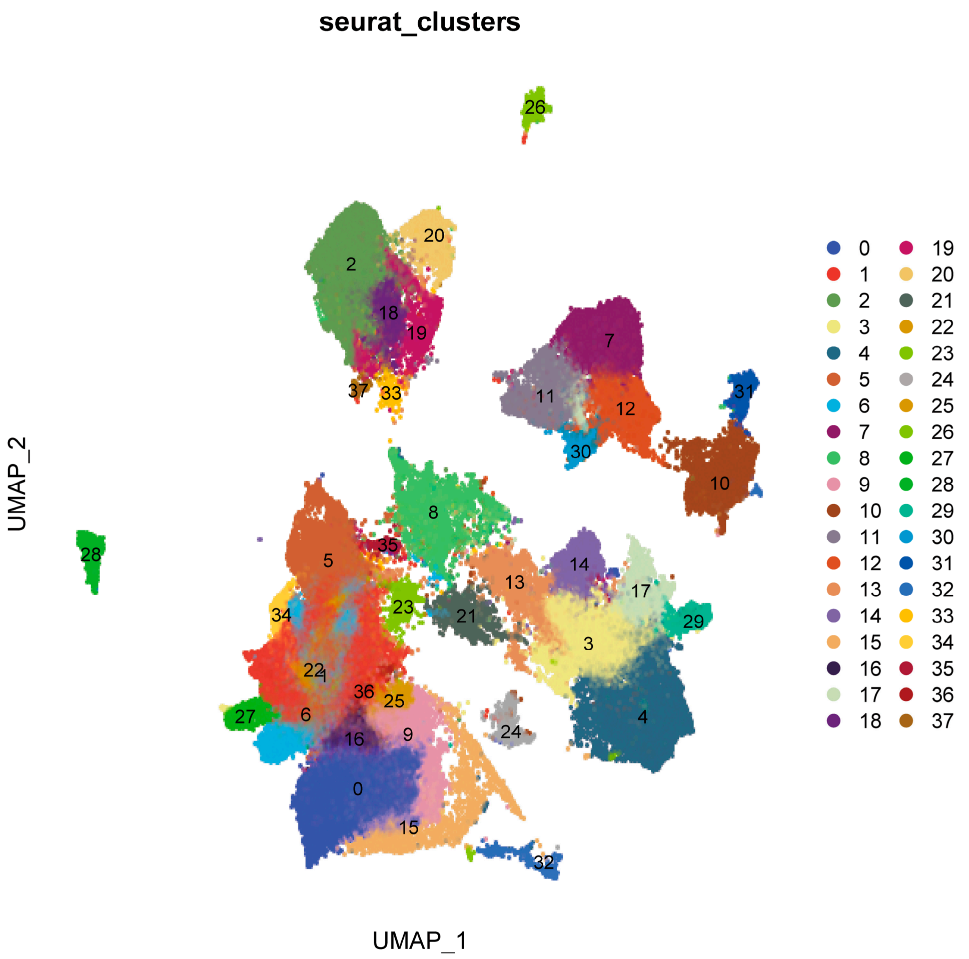

Figure S3 The UMAP dimensionality reduction graph of the primitive clusters in OCSCDs.

Figure S4

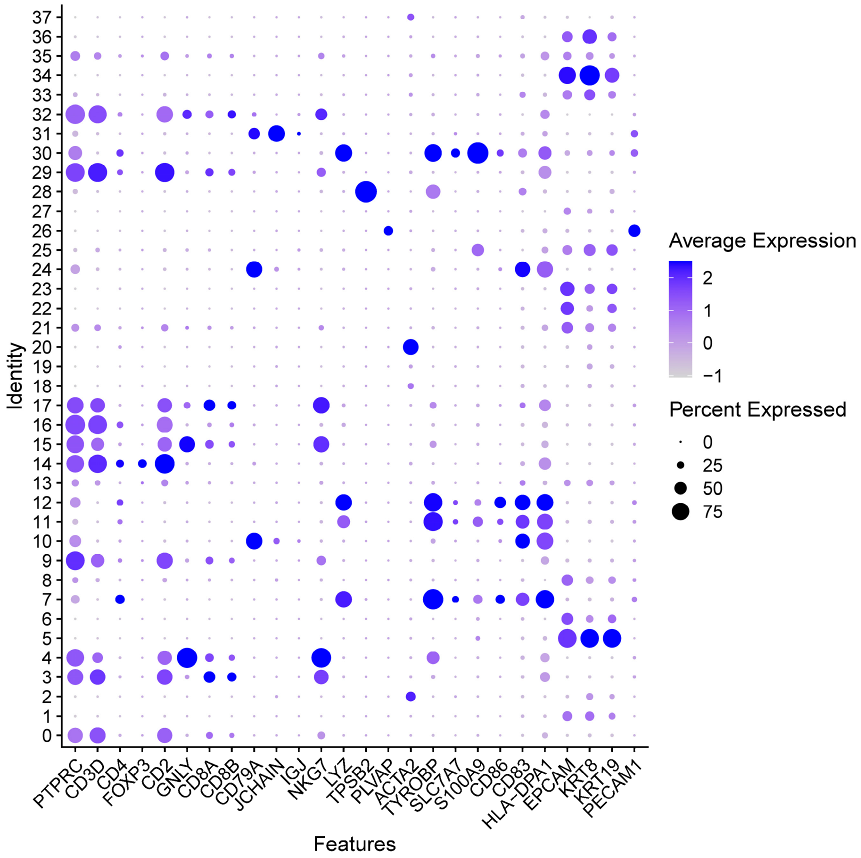

Figure S4 The expression levels of marker genes in different clusters of OCSCDs.

Table S1 The primer sequence used in this study.

Gene	GenBank Accession	Sequence（5’- 3’）
FOXP3	NM_002046.7	F ACACTGCOCCTAGTCATGGT
		R CGTTGAGAGCTGGTGCATGA
TGF-β	NM_000660	F CTAATGGTGGAAACCCACAACG
		R TATCGCCAGGAATTGTTGCTG
IL10	NM_000572.3	F GACTTTAAGGGTTACCTGGGTTG
		R TCACATGCGCCTTGATGTCTG
GAPDH	NM_001256799	F TCGGAGTCAACGGATTTGGT
		R TTCCCGTTCTCAGCCTTGAC
